# Cervical carcinogenesis risk association of HPV33 ***E6*** and ***E7*** genetic variations in Taizhou, Southeast China

**DOI:** 10.1186/s12985-023-02125-9

**Published:** 2023-07-19

**Authors:** Zi-Yi Yan, Xing-Hong Di, Yi Qiu, Yuan-Yuan Ying, Jun Gan, Hui-Hui Xu

**Affiliations:** 1 Medical Research Center, Taizhou Hospital of Zhejiang Province, Wenzhou Medical University, Linhai, 317000 Zhejiang China; 2 Key Laboratory of Minimally Invasive Techniques & Rapid Rehabilitation of Digestive System Tumor of Zhejiang Province, Linhai, Zhejiang China; 3 Scientific Research Department, Taizhou Hospital of Zhejiang Province, Wenzhou Medical University, Linhai, Zhejiang China

**Keywords:** Human papillomavirus 33, Genetic variability, Phylogenetic analysis, Cervical cancer

## Abstract

**Background:**

Human papillomavirus (HPV) 33 belongs to the Alphapapillomavirus 9 (α-9 HPV) species group, which also contains types 16, 31, 35, 52, 58 and 67. The purpose of this study was to investigate the genetic variations of HPV33 and to explore its carcinogenicity among women in Taizhou, Southeast China.

**Methods:**

Exfoliated cervical cells were collected for HPV genotyping. Only single HPV33 infection cases were selected, and their *E6* and *E7* genes were sequenced using the ABI 3730xl sequencer and then analysed using MEGA X.

**Results:**

From 2014 to 2020, a total of 185 single HPV33-positive specimens were successfully amplified. We obtained 15 distinct HPV33 E6/E7 variants, which were published in GenBank under accession numbers OQ672665-OQ672679. Phylogenetic analysis revealed that all HPV33 E6/E7 variants belonged to lineage A, of which 75.7% belonged to lineage A1. Compared with CIN1, the proportion of sublineage A1 in CIN2/3 was higher, but there was no significant difference (76.5% vs. 80.6%, *P* > 0.05). Altogether, 20 single nucleotide substitutions were identified, of which 6 were novel substitutions, including T196G (C30G), A447T, G458T (R117L), G531A, A704A, and C740T. In addition, no significant trends were observed between the nucleotide substitutions of HPV33 E6/E7 variants and the risk of cervical lesions.

**Conclusion:**

This study provides the most comprehensive data on genetic variations, phylogenetics and carcinogenicity of HPV33 E6/E7 variants in Southeast China to date. The data confirmed that cervical lesions among women in Taizhou are attributable to HPV33, which may be due to the high infection rate of sublineage A1 in the population.

**Supplementary Information:**

The online version contains supplementary material available at 10.1186/s12985-023-02125-9.

## Background

Human papillomavirus (HPV) is the most common pathogen of cervical cancer, especially the high-risk HPV types [1]. HPV16 and 18 are the two most carcinogenic types, accounting for approximately 70% of cervical cancers worldwide [2]. Types 31, 33, 35, 39, 45, 51, 52, 56, 58, 59, 66, and 68 are related to the remaining cases of cervical cancers. Notably, HPV type distribution varies with geographical location and ethnic group. Moreover, these types differ in biological functions due to differences in genetic variations, which may become a key risk factor in cervical cancer.

Based on the American Society for Colposcopy and Cervical Pathology (ASCCP) guidelines, colposcopy is recommended immediately in women with HPV16 or 18 infections [3]. However, our previous study showed that women infected with HPV16, 18, 31, 33, 52, or 58 should be recommended for colposcopy immediately, which can improve the detection rate of CIN2 + lesions in the Taizhou area of southeast China [4]. HPV types 33, 52 and 58 were detected less frequently than HPV16 in terms of association with severe cervical intraepithelial neoplasia (CIN) lesions but more frequent than HPV18 in China [4, 5]. Notably, HPV33 has a higher absolute risk for CIN2 + than other non-HPV16 high-risk types [6–8]. Therefore, our research team should devote more attention to these common carcinogenic types, such as HPV33.

HPV33 was originally cloned from cervical cancer in 1986 [9]. HPV33 variants are clustered into three main phylogenetic lineages (A, B, and C) and five sublineages (A1, A2, A3, B1, and C1) [10, 11]. These lineages exhibit a distribution pattern that varies across the world. Unfortunately, the data available on HPV33 genetic variations and their carcinogenicity are still limited in China. Therefore, the purpose of this study was to investigate the genetic variations in the *E6* and *E7* oncogenes of HPV33 and to explore their potential role in cervical cancer risk among Chinese women in the Taizhou area.

## Materials and methods

### Study population and specimen collection

From February 2014 to December 2020, exfoliated cervical cells were collected from women who underwent cervical screenings at Taizhou Hospital, Zhejiang Province. Subsequently, HPV genotyping was performed, which has been described in detail in our previous study [4]. Only single HPV33 infection cases were selected for this study. The specimens were stored in cell preservation buffer at -20 °C.

### PCR and sequencing

DNA was extracted from stored specimens using a DNA Extraction Kit (#GK0122, GENEray, China) according to the manufacturers’ guidelines. Specific primer pairs were designed for the entirety of the E6 and E7 regions of HPV33 using the NCBI Primer-BLAST tool. The primers were as follows: 33E6E7F 5’-AGGGTGTAACCGAAAGCGG-3’ and 33E6E7R 5’-TTGCAGCACGATCAACAACG-3’. The PCR system and conditions were described in our previous HPV study [12]. The length of the PCR product was 1164 bp (nucleotide sites [nt] 31-1175, including the *E6* gene nt 109–558 and *E7* gene nt 573–866). Subsequently, PCR products were purified and sequenced at BGI company (Hangzhou, China), and all data were confirmed by repeating PCR and sequencing reactions at least twice.

### Phylogenetic analysis

All acquired nucleotide sequences were aligned by BioEdit software using the reference sequence (GenBank accession no. M12732.1) as a standard for HPV33 nucleotide position numbering. The phylogenetic tree was generated using 15 complete HPV33 *E6*/*E7* genes obtained in this study and 6 complete HPV33 E6/E7 genes available in NCBI (GenBank accession no. M12732.1 (A1), HQ537697 (A1), HQ537698 (A2), EU918766 (A3), HQ537705 (B1), and KF436865 (C1)). A maximum-likelihood tree was constructed by MEGA X software, with one thousand bootstrap replicates.

### Statistical analysis

All analyses were performed using SPSS 16.0 software. The association of cervical lesion risk with HPV33 E6/E7 variants was analysed using the chi-square test or Fisher’s exact test. A two-sided *P* value < 0.05 was regarded as statistically significant.

## Results

### Characteristics of the study population

From February 2014 to December 2020, a total of 189 women with a single HPV33 infection were selected. The average age was 42.3 years (range 19–73 years). A total of 185 (97.9%) sequences of the entire *E6* and *E7* genes from HPV33 were successfully obtained. Due to the small number of HPV copies, the remaining 4 (2.1%) sequences were excluded. Out of 185 cases, 110 (59.5%) underwent colposcopy biopsy for histological diagnosis, including 57 with normal cervices after biopsy, 17 with CIN1, 16 with CIN2, 20 with CIN3, and no cases of cervical cancer. The characteristics of the study population are shown in Table 1.

**Table 1:** Characteristics of the study population categorized by HPV33 (sub)lineage

HPV52 (sub)lineages	Normal (n = 57)	CIN1(n = 17)	CIN2(n = 16)	CIN3(n = 20)	Cancer (n = 0)	Total (n = 110)
**A1**	**39(68.4%)**	**13(76.5%)**	**12(75.0%)**	**17(85.0)**	**0**	**81(73.6%)**
33CNTZ01	36(63.2%)	12(70.6%)	11(68.8%)	15(75.0%)	0	74(67.3%)
33CNTZ02	0	1(5.9%)	1(6.3%)	1(5.0%)	0	3(2.7%)
33CNTZ05	1(1.8%)	0	0	0	0	1(0.9%)
33CNTZ08	1(1.8%)	0	0	1(5.0%)	0	2(1.8%)
33CNTZ09	1(1.8%)	0	0	0	0	1(0.9%)
**A2**	**0**	**0**	**1(6.3%)**	**0**	**0**	**1(0.9%)**
33CNTZ10	0	0	1(6.3%)	0	0	1(0.9%)
**A3**	**18(31.6%)**	**4(23.5%)**	**3(18.7%)**	**3(15.0%)**	**0**	**28(25.5%)**
33CNTZ11	13(22.8%)	3(17.6%)	3(18.7%)	3(15.0%)	0	22(20.0%)
33CNTZ12	1(1.8%)	1(5.9%)	0	0	0	2(1.8%)
33CNTZ13	2(3.5%)	0	0	0	0	2(1.8%)
33CNTZ14	1(1.8%)	0	0	0	0	1(0.9%)
33CNTZ15	1(1.8%)	0	0	0	0	1(1.8%)

### Variations in the ***E6*** and ***E7*** genes

**Fig. 1:**
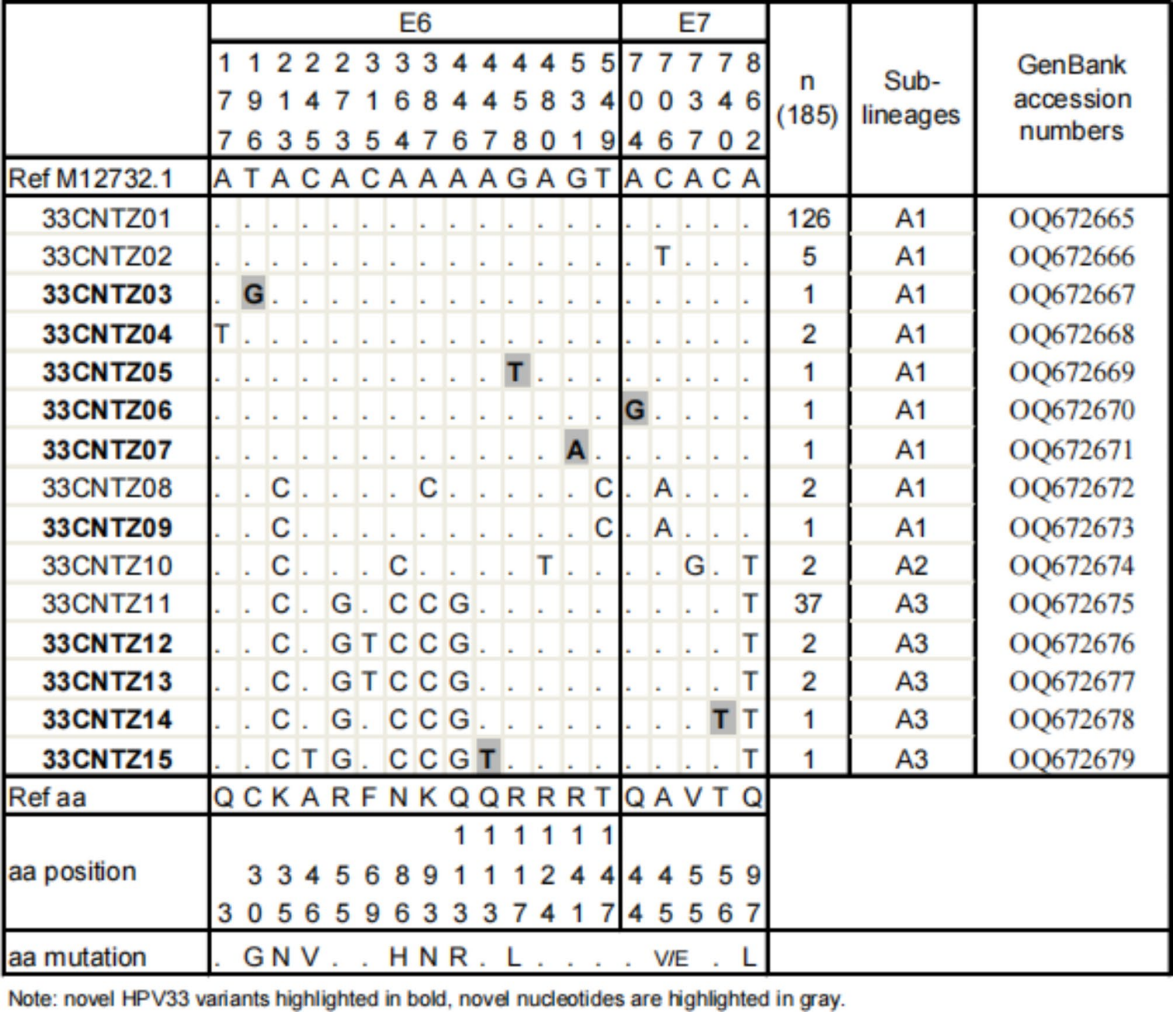
Genetic variability of HPV33 *E6* and *E7* nucleotide sequences in Taizhou area, Southeast China. Numbering refers to the first nucleotide of the HPV33 prototype reference sequence (GenBank: M12732.1). Each row indicates the variant identification and the nucleotide sequence alignment compared to the reference. Novel HPV33 variants are highlighted in bold and novel nucleotide substitutions are highlights in gray

Compared with M12732.1, 59 of the 185 HPV33 samples (31.9%) showed nucleotide variations. Figure 1 visually distinguishes all changes in nucleotide and amino acid sequences in the entire *E6* and *E7* fragments of the HPV33 lineage/sublineages. In this study, 15 distinct variation patterns were detected in the HPV33 E6/E7 variants, denoted 33CNTZ01-33CNTZ15, which were published with GenBank accession nos. OQ672665 to OQ672679. 33CNTZ01 (68.1%, 126/185) was the most common variant in the Taizhou-based population, with complete E6 and E7 sequence homology with M12732.1. Ten (66.7%, 10/15) belonged to the novel HPV33 E6/E7 variants, which are highlighted in bold in Fig. 1.

Altogether, 20 single nucleotide substitutions were identified, with 6 (30.0%) novel substitutions and 10 (50.0%) nonsynonymous substitutions. Nonsynonymous substitutions included T196G (C30G), A213C (K35N), C245T (A46V), A364C (N86H), A387C (K93N), A446G (Q113R), and G458T (R117L) in the E6 sequence and C706T (A45V), C706A (A45E), and A862T (Q97L) in the E7 sequence. To our knowledge, the nucleotide substitutions of T196G (C30G), A447T, G458T (R117L), G531A, A704A, and C740T have not been reported in previous studies.

### Phylogenetic construction

**Fig. 2:**
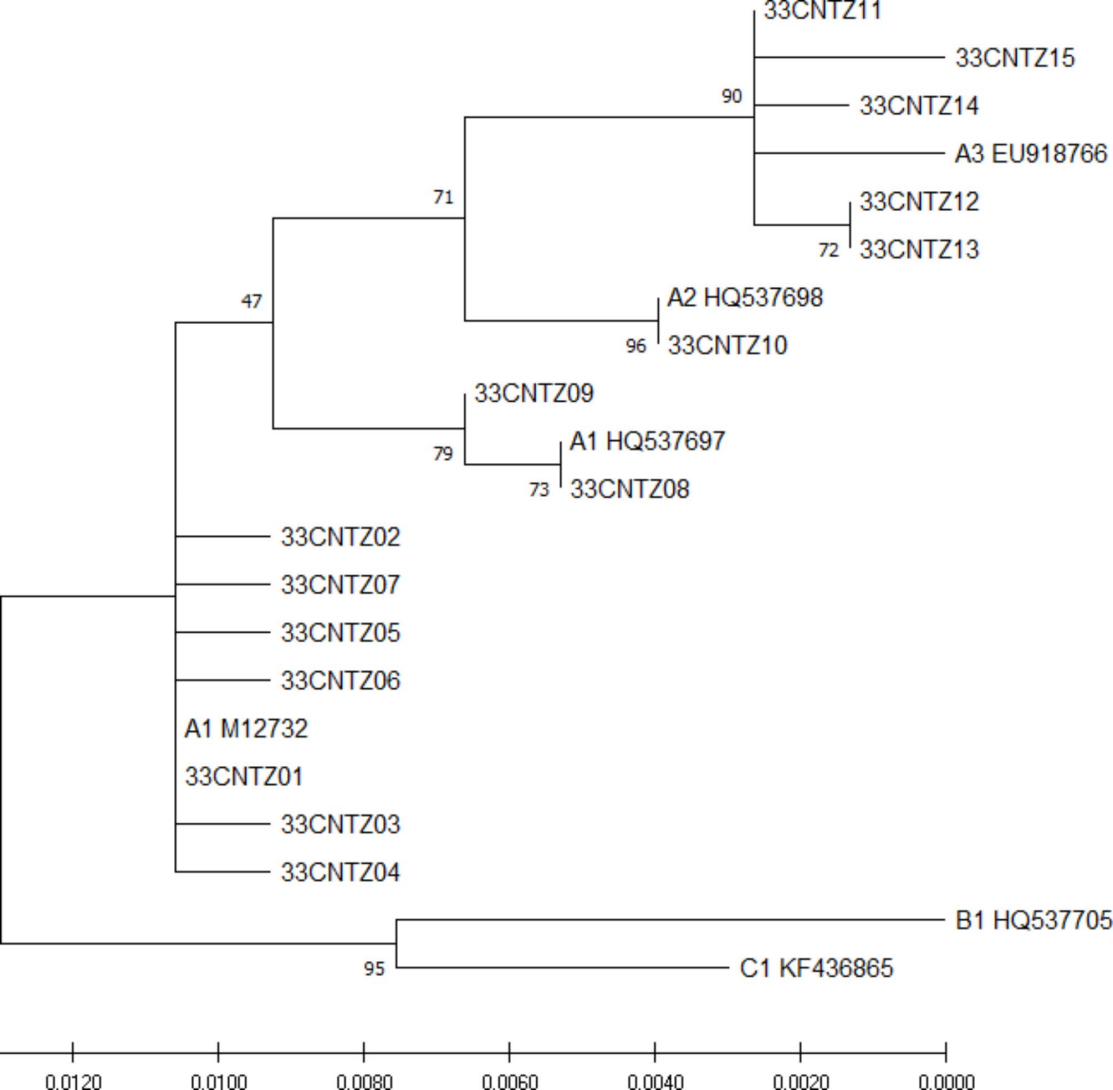
Phylogenetic tree of the HPV33 E6/E7 variants. Maximum-likelihood analysis (with MEGA X) of *E6* and *E7* nucleotide sequences was inferred from 15 obtained HPV33 E6/E7 variants and 5 reference sequences. Numbers below branches indicate bootstrap values

The phylogenetic tree was constructed from 21 complete HPV33 E6 and E7 sequences (15 obtained from our study and 6 from GenBank) (Fig. 2). Based on the phylogenetic tree, sublineages A1, A2, and A3 were detected in 75.7% (140/185), 1.1% (2/185), and 23.2% (43/185) of samples, respectively. All HPV33 E6/E7 variants belonged to lineage A, whereas no variants belonged to lineage B or lineage C in the present study.

### Risk association with cervical lesions

Our data suggested that the proportion of sublineage A1 in CIN2/3 was higher when compared with CIN1, but it was not significant (76.5% vs. 80.6%, *P* > 0.05). There was no significant difference in the risk of cervical lesions between sublineage A1 and other HPV33 E6/E7 variants. No significant difference was observed in the relative risk for cervical lesions among the nucleotide variations in the HPV33 *E6* and *E7* genes (Table 1).

## Discussion

Cervical cancer remains a serious public health problem in developing countries, especially in China. The distribution of HPV types may differ among specific populations in different countries. Their genetic variation data can be of considerable importance to assess the impact of local HPV vaccines and strategies for cancer prevention. To our knowledge, the Taizhou Area HPV Study is a comprehensive study to assess the genetic variations of HPV types in southeast China, which is helpful for local HPV vaccine development. According to our previous findings, genetic variation in the *E6* and *E7* genes of α-9 HPV types was found to be highly associated with cervical carcinogenesis risk [13–15]. Therefore, we continue to investigate the genetic variation of HPV33 and to explore its carcinogenicity among women in Taizhou, Southeast China.

In this study, we obtained 185 complete sequences of HPV33 *E6* and *E7* genes among Chinese women. Our data showed that all HPV33 E6/E7 variants belonged to lineage A. Most of the HPV33 E6/E7 variants (75.7%) belonged to sublineage A1, which was similar to the results (92.1%) obtained in Southwest China [16]. Sublineage A1 was observed to be distributed throughout the world, but the relative frequency varied by region. Sublineage A2 (59.7%) was the most common lineage in the Asia and Oceania region [10]. However, only 1.1% of the HPV33 variants belonged to A2, and 23.2% of the sublineage belonged to A3 in Taizhou, Southeast China. Lineage A predominates in Asia and Europe, while lineage B or C predominates in Africa [10, 17]. Compared with other sublineage variants, the risk of CIN2/3 or cervical cancer in sublineage A1 was significantly increased [18–20]. Our data suggested that the proportion of sublineage A1 in CIN2/3 was higher when compared with CIN1, but it was not significant (76.5% vs. 80.6%, *P* > 0.05). There was no significant difference in the risk of cervical lesions between sublineage A1 and other HPV33 E6/E7 variants. Notably, 33CNTZ01 accounts for 90% (126/140) of A1 and 33CNTZ11 accounts for 86% (37/43) of A3. Other (sub)lineages were not included in these analyses due to having only one or two samples.

Compared with the HPV33 reference sequence (GenBank accession no. M12732.1), the six most prevalent nucleotide substitutions were A213C (K35N) (25.9%), A364C (N86H) (24.3%), A273G (23.2%), A387C (K93N) (24.3%), and A446G (Q113R) (23.2%) in the *E6* gene and A862T (Q97L) (24.3%) in the *E7* gene, which were specific to HPV33 lineage A3. He et al. [21, 22] found 15.3% G542T (R145I) of the *E6* gene in Southwest China; however, this nucleotide substitution has not been detected in Taizhou, Southeast China. It is hypothesized that the substitution of G542T(R145I) may increase the infection rate of the HPV33 variant in Southwest China by reducing its immunogenicity and enhancing its adaptability to the environment [22]. In addition, the 93rd residue is the common nonsynonymous substitution in the *E6* gene of α-9 HPV types [14, 15, 22]. These findings are valuable for the development of therapeutic vaccines and cancer immunotherapies.

## Conclusions

In summary, this study provides the most comprehensive data on the genetic variations, phylogenetics, and carcinogenicity of HPV33 E6/E7 variants in Southeast China to date. These findings can make an important contribution to future epidemiological, HPV vaccination, and cancer immunotherapy studies.

## Electronic supplementary material

Below is the link to the electronic supplementary material.

**Additional file 1.** Clinical data for HPV33 study in Taizhou area, China

## Data Availability

All data generated during this study are included in this published article. The supplementary materials included the nucleotide variations of the *E6* and *E7* genes from HPV33 and the follow-up data of patients. In addtion, these sequences have been released to GenBank database with the accession codes of OQ672665-OQ672679. The links are https://www.ncbi.nlm.nih.gov/nuccore/OQ672665 ~ https://www.ncbi.nlm.nih.gov/nuccore/OQ672679.

## Abbreviations

bp: Base pair
CIN: Cervical intraepithelial neoplasia
DNA: Deoxyribonucleic acid
HPV: Human papillomavirus
LCR: Long control region
LEEP: Loop electrosurgical excision procedure
nt: Nucleotide sites
OR: Odds ratios
ORF: Open reading frames
PCR: Polymerase chain reaction
SNP: Single nucleotide polymorphism
WHO: World Health Organization
